# Zinc levels in seminal plasma and their correlation with male infertility: A systematic review and meta-analysis

**DOI:** 10.1038/srep22386

**Published:** 2016-03-02

**Authors:** Jiang Zhao, Xingyou Dong, Xiaoyan Hu, Zhou Long, Liang Wang, Qian Liu, Bishao Sun, Qingqing Wang, Qingjian Wu, Longkun Li

**Affiliations:** 1 Department of Urology, Second Affiliated Hospital, Third Military Medical University, Chongqing, 400037, China

## Abstract

Zinc is an essential trace mineral for the normal functioning of the male reproductive system. Current studies have investigated the relationship between seminal plasma zinc and male infertility but have shown inconsistent results. Hence, we systematically searched PubMed, EMBASE, Science Direct/Elsevier, CNKI and the Cochrane Library for studies that examined the relationship between seminal plasma zinc and male infertility, as well as the effects of zinc supplementation on sperm parameters. Twenty studies were identified, including 2,600 cases and 867 controls. Our meta-analysis results indicated that the seminal plasma zinc concentrations from infertile males were significantly lower than those from normal controls (SMD (standard mean differences) [95% CI] −0.64 [−1.01, −0.28]). Zinc supplementation was found to significantly increase the semen volume, sperm motility and the percentage of normal sperm morphology (SMD [95% CI]: −0.99 [−1.60, −0.38], −1.82 [−2.63, −1.01], and −0.75 [−1.37, −0.14], respectively). The present study showed that the zinc level in the seminal plasma of infertile males was significantly lower than that of normal males. Zinc supplementation could significantly increase the sperm quality of infertile males. However, further studies are needed to better elucidate the correlation between seminal plasma zinc and male infertility.

Infertility is defined as the lack of ability to conceive within one year of unprotected intercourse with the same partner^1^. It is estimated that nearly 8–12% of couples are infertile^2^, and approximately 30–40% of infertility cases are caused by male factors^3^. Several risk factors are involved in the pathogenesis of infertility, some of which include alterations in spermatogenesis due to testicular cancer, aplasia of the germinal cells, varicocele, defects in the transport of sperm, or environmental factors as well as congenital anomalies, infectious diseases, bilateral spermaducts, pregnancy-related infections, alterations in the characteristics of semen such as a decrease in sperm motility and sperm count, the presence of antisperm antibodies (ASAs), and nutritional deficiency of trace elements such as selenium and zinc (Zn)^4^,^5^,^6^,^7^,^8^,^9^.

Trace elements play an important role in the male reproductive process because of their high activity at the molecular level, although they are known to exist in the body at very low levels. Zn is second only to iron as the most abundant element in human tissues. Although Zn is found in most types of foods such as red meat, white meat, fish, and milk, the World Health Organization (WHO) estimates that one-third of the world’s population is deficient in zinc. Zinc and citrate are excreted from the prostate gland as a low-molecular-weight complex; thus, it is estimated that the zinc levels in seminal plasma typically represent prostatic secretory function. After ejaculation, half of the quantity of this complex is redistributed and linked to medium- and high-molecular-weight compounds generated from the seminal vesicles^9^. The decrease in the seminal plasma zinc concentration may result in inadequate intake, reduced absorption, increased losses, or increased demand. Additionally, the commonest worldwide cause is inadequate intake as a result of a diet low in Zn or rich in phytate. Additionally, increased urinary losses can occur under conditions associated with muscle catabolism, such as sepsis, or iatrogenically from the prolonged use of drugs^10^,^11^. Furthermore, some studies have reported that a sharp decrease in zinc in the prostatic fluid must result in a decreased zinc concentration in seminal plasma^11^,^12^,^13^.

During reproduction, zinc has numerous important functions, and it is essential for conception, implantation, and a favorable pregnancy outcome^10^,^14^. Zinc is present in high concentrations in the seminal fluid, and it could play a multifaceted role in sperm functional properties. It influences the fluidity of lipids and, thus, the stability of biological membranes^15^. It affects the stability of sperm chromatin^16^. It is involved in the formation of free oxygen radicals^17^, and it could play a regulatory role in the process of capacitation and the acrosome reaction^18^. However, little is known concerning the role of zinc in seminal plasma or serum regarding the global functional competence of human spermatozoa, such as the sperm’s ability to penetrate cervical mucus (CM) or its fertilizing capacity. The relationship of zinc to the routinely determined variables of semen quality has been controversial^19^,^20^,^21^.

Current studies have investigated the correlations between seminal plasma zinc concentrations and male infertility but have shown contradictory results, with some studies showing that the seminal plasma zinc concentrations of infertile men were significantly lower than those of normal controls; however, other studies did not report this outcome^22^,^23^,^24^,^25^,^26^,^27^,^28^,^29^,^30^,^31^,^32^,^33^,^34^,^35^,^36^,^37^,^38^,^39^,^40^,^41^. Moreover, some studies have reported that zinc supplementation in the treatment of infertility could significantly increase the sperm quality of infertile males, while other studies have shown opposing results^25^,^26^,^28^,^30^,^31^,^37^. Therefore, we systematically reviewed the available literature and performed a meta-analysis to evaluate the correlations between seminal plasma zinc concentrations and male infertility and the effects of zinc supplementation on sperm parameters to possibly provide valuable insights into the diagnosis and treatment of male infertility.

## Results

### Characteristics of the included studies

**Figure 1:**
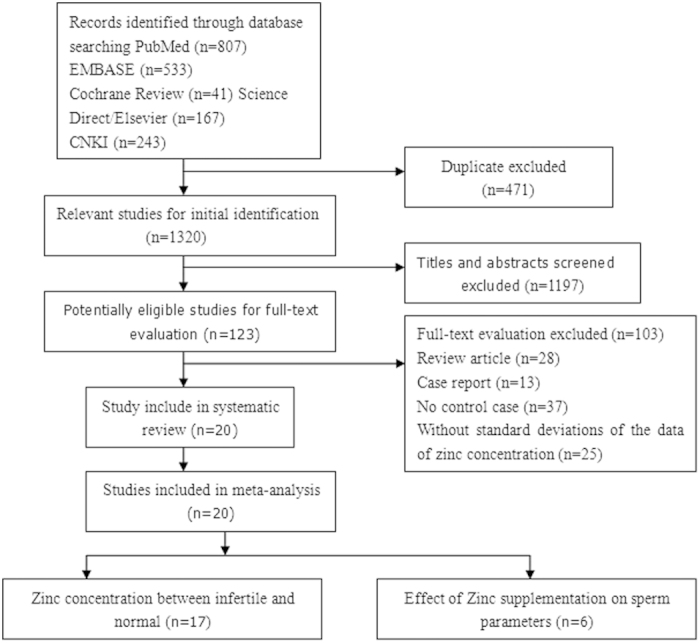
Flow diagram of the selection of eligible studies.

Figure 1 shows the detailed review process. In total, 1,320 unduplicated studies were identified, and twenty studies were ultimately selected according to the eligibility criteria. After group discussion, all of the reviewers were in agreement to include all twenty papers. Table 1 summarizes the general data from the eight studies. The retrieved studies involved 2,600 infertile males and 867 normal controls. The age ranges of the patient and control groups were 29.2–49.3 years and 30.9–36.6 years, respectively. The mean ages of the patient and control groups were unavailable for thirteen studies^23^,^24^,^26^,^27^,^29^,^32^,^34^,^36^,^37^,^38^,^39^,^40^,^41^. All of these studies reported exclusion/inclusion criteria^22^,^23^,^24^,^25^,^26^,^27^,^28^,^29^,^30^,^31^,^32^,^34^,^35^,^36^,^37^,^38^,^39^,^40^,^41^. Thirteen of 20 studies included the abstinence time before semen collection^23^,^25^,^30^,^32^,^33^,^34^,^35^,^36^,^37^,^38^,^39^,^40^,^41^. Of the seventeen studies^22^,^23^,^24^,^25^,^28^,^30^,^31^,^32^,^34^,^35^,^36^,^37^,^39^,^40^,^41^ that studied the correlation of seminal plasma zinc concentrations with male infertility (Table 2), 1,893 infertile males and 792 normal controls were included. Six studies^26^,^27^,^29^,^31^,^32^,^38^ studied the effects of zinc supplementation on sperm parameters and included 563 infertile males.

## Meta-analysis

### Seminal plasma zinc concentration between normal and infertile males

**Figure 2:**
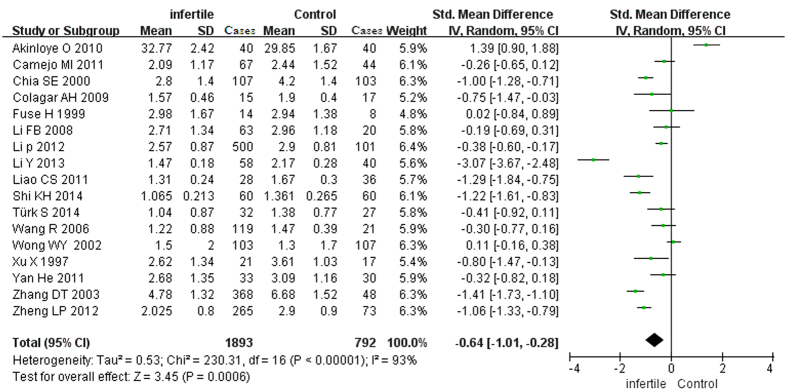
Forest plot showing the meta-analysis outcomes of the plasma zinc between infertile and normal men. Abbreviations: IV: inverse variance; Random: random-effects model.

**Figure 3:**
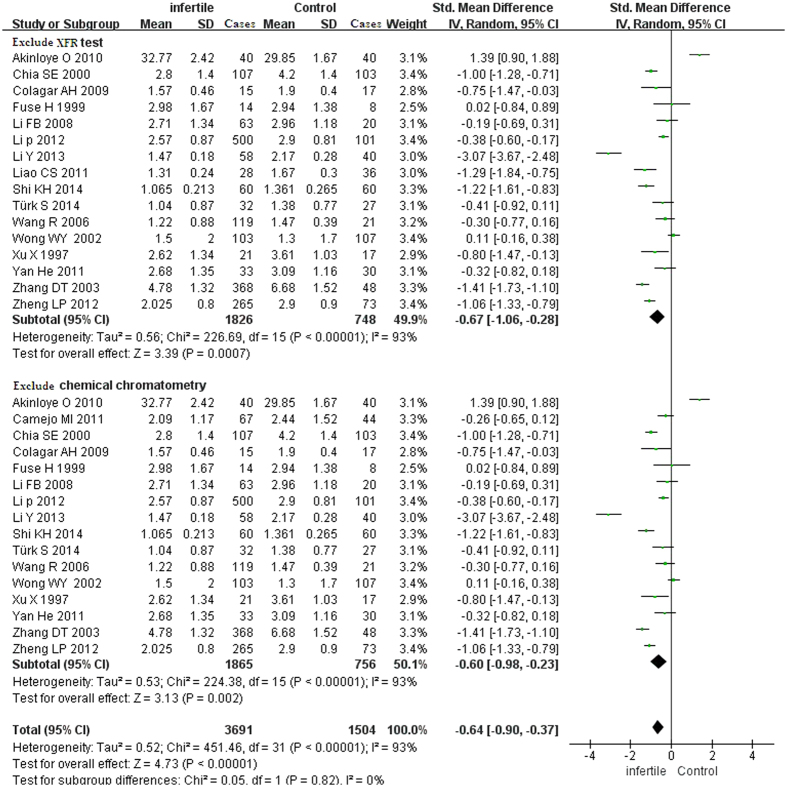
Sub-group forest plot showing the meta-analysis outcomes of seminal plasma zinc between infertile and normal men. Abbreviations: IV: inverse variance; Random: random-effects model. Exclude XFR test: studies only included AAS and the chemical chromatometry test. Exclude chemical chromatometry test: studies only include AAS and XRF test.

The test of heterogeneity suggested a random-effects model, and the meta-analysis revealed that the seminal plasma zinc concentrations from infertile patients were significantly lower than those from normal controls (SMD [95% CI]: −0.64 [−1.01, −0.28]) (Fig. 2). Because one study detected the zinc concentration by XFR (radionuclide-induced energy dispersive X-ray fluorescence), and two studies detected the zinc concentration by chemical chromatometry testing, we also conducted a sub-analysis that excluded XFR and chemical chromatometry testing; however, the final conclusion was not changed (the seminal zinc concentrations from the infertile patients were significantly lower than those from the normal controls) (Fig. 3).

### Effect of zinc supplementation on sperm parameters

**Figure 4:**
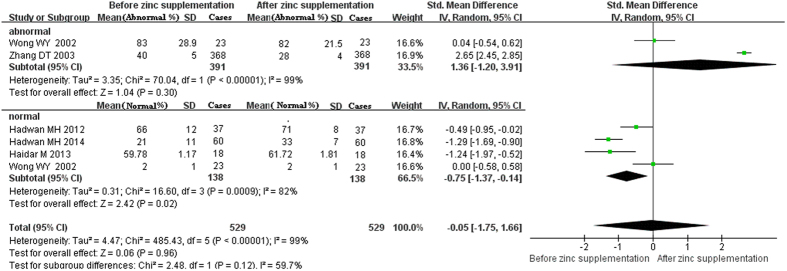
Forest plot showing the meta-analysis outcomes of the effect of zinc supplementation on abnormal and normal sperm morphology. Abbreviations: IV: inverse variance; Random: random-effects model. Normal (%): percent of normal morphology; abnormal (%): percent of abnormal morphology.

**Figure 5:**
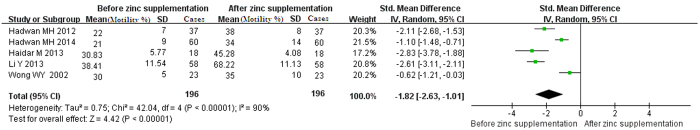
Forest plot showing the meta-analysis outcomes of the effect of zinc supplementation on sperm motility. Abbreviations: IV: inverse variance; Random: random-effects model.

**Figure 6:**
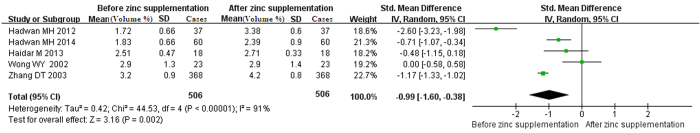
Forest plot showing the meta-analysis outcomes of the effect of zinc supplementation on the semen volume. Abbreviations: IV: inverse variance; Random: random-effects model.

**Figure 7:**
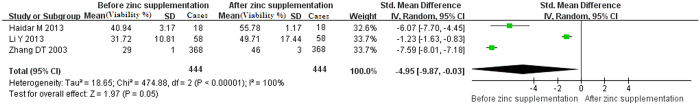
Forest plot showing the meta-analysis outcomes of the effect of zinc supplementation on sperm viability. Abbreviations: IV: inverse variance; Random: random-effects model.

**Figure 8:**
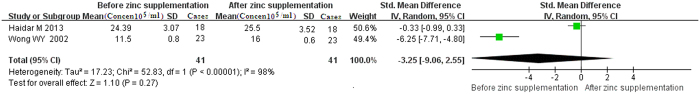
Forest plot showing the meta-analysis outcomes of the effect of zinc supplementation on sperm concentration. Abbreviations: IV: inverse variance; Random: random-effects model.

**Figure 9:**
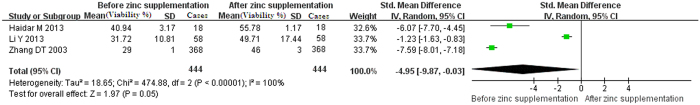
Forest plot showing the meta-analysis outcomes of the effect of zinc supplementation on the sperm count. Abbreviations: IV: inverse variance; Random: random-effects model.

For the curative effects of zinc supplementation in the treatment of male infertility, the meta-analysis revealed that zinc supplementation can significantly increase the percentage of normal sperm morphology, sperm motility and semen volume (SMD [95% CI]: −0.75 [−1.37, −0.14], −1.82 [−2.63, −1.01], and −0.99 [−1.60, −0.38], respectively) (Figs 4, 5, 6). However, there were no significant effects of zinc supplementation on the sperm viability, sperm concentration, sperm count or percentage of abnormal sperm morphology (Figs 4 and 7, 8, 9).

### Publication bias of the included studies

**Figure 10:**
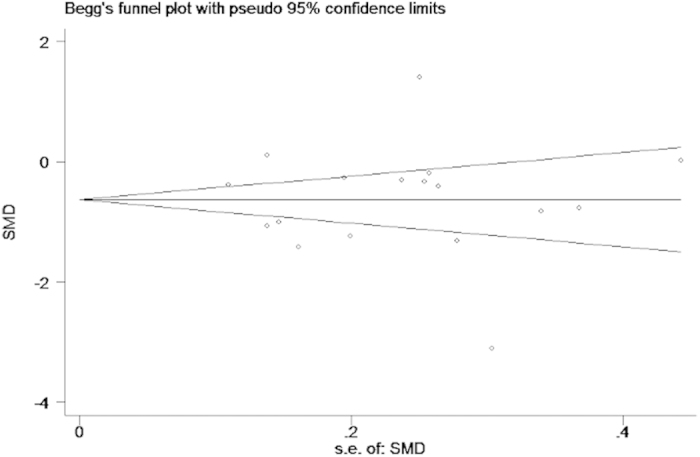
Begg’s publication bias plot of the seminal plasma zinc concentration between infertile and normal men. The funnel plot did not show any substantial asymmetry, suggesting no evidence of publication bias.

Begg’s funnel plot showed no substantial asymmetry (Fig. 10). Egger’s regression test of publication bias of the seminal plasma zinc in infertile and normal males indicated little evidence of publication bias (t = −0.19 P = 0.85 > 0.05) (Table 3).

### Sensitivity analysis of the meta-analysis

**Figure 11:**
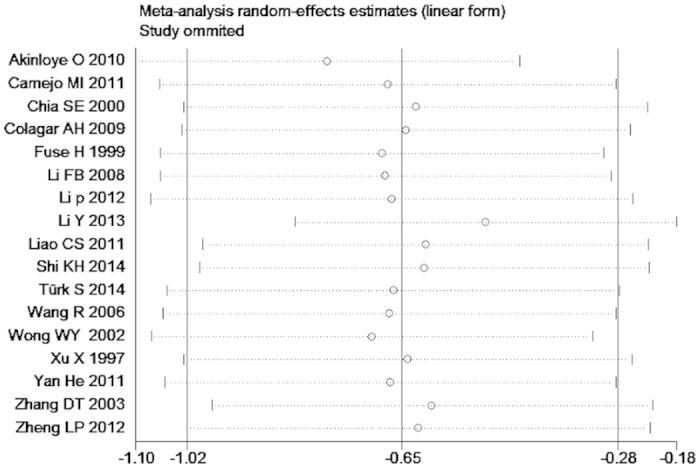
Sensitivity analysis plot of the seminal plasma zinc concentration between infertile and normal men.

We omitted one study sequentially, and the calculated combined SMD for the remaining studies yielded consistent results. In the overall meta-analysis, no single study significantly changed the combined results, indicating that the results were statistically stable and reliable (Fig. 11).

## Discussion

In our study, seventeen articles studied the correlation between seminal plasma zinc concentrations and male infertility. Nine studies reported that the zinc concentrations in seminal plasma from infertile men were significantly lower than those from normal men^24^,^30^,^32^,^34^,^35^,^37^,^38^,^39^,^40^; one study reported that the zinc concentration in the seminal plasma from infertile men was significantly higher than that in normal men^28^, and the other seven studies showed no significant difference between infertile and normal males^22^,^23^,^24^,^31^,^33^,^36^,^41^. In this meta-analysis, the zinc concentrations in seminal plasma from infertile males were significantly lower than those in normal males. Six of twenty articles studied the curative effects of zinc supplementation in the treatment of male infertility and its effect on sperm parameters. Our results revealed that zinc supplementation could significantly increase the sperm volume, sperm motility and percentage of normal sperm morphology of infertile men. After zinc supplementation, the sperm quality of infertile men was significantly increased.

The concentration of zinc in human seminal plasma is higher than that in other tissues^42^. In fact, during the early stages of sperm development, spermatogenic cells reside within the seminiferous tubules, which have a Zn content similar to or lower than that of other organs, such as the liver or kidneys. Subsequently, sperm encounter, in succession, the epididymis, vas deferens and seminal vesicles, which are characterized by a progressively increased tissue Zn content^43^,^44^. Finally, spermatozoa are ejaculated into seminal plasma, which is essentially formed by prostate secretions in which Zn is nearly 100 times more concentrated than in blood serum. Foresta C also suggested that, along the entire genital tract, there is a prevalent expression of Zn transporters that supply Zn. Additionally, from the germ cells to mature sperm, there is an overall uptake of Zn, and, before ejaculation, the prostate secretions concur to stabilize sperm^10^. All of these mechanisms are a prerequisite for mature sperm to be able to undergo capacitation, motility hyperactivation and the acrosome reaction when the Zn levels fall during their transit in the female genital tract.

In the human reproductive system, Zn plays an important role in spermatogenesis, from its formation and contribution to the ultrastructural stabilization of chromatin compaction to the modulation of mitochondria-dependent processes, such as cell respiration and programmed cell death^45^,^46^. Zinc is a metalloprotein cofactor for DNA-binding proteins with Zn fingers. It is part of copper (Cu)/zinc superoxide dismutase, and several proteins are involved in the repair of damaged DNA and transcription and translation processes of DNA^47^,^48^.

Several studies have investigated the curative effects of zinc supplementation in the treatment of male infertility and its effects on sperm parameters, but they have shown inconsistent results. There are several mechanisms by which zinc might interfere with sperm function. First, zinc is a cofactor for several hundred metalloenzymes, particularly the enzymes responsible for protein synthesis^49^,^50^,^51^,^52^. It influences phospholipases^53^, thus modulating the stability of biological membranes. It has been suggested that the removal of zinc from the sperm cell surface destabilizes the plasma membrane, playing an important role in preparation for the completion of capacitation and the acrosome reaction. Some studies have reported that zinc supplementation can also improve the synthesis of metallothioneins (low-molecular-weight Zn-binding proteins), which have properties of enhancing the quality of seminal fluids to protect sperm against damage^54^; metallothioneins have the property of protecting biological tissues from the damage of oxidative stress via the capture of harmful oxidant species, such as superoxide and hydroxyl radicals^55^. Second, zinc in seminal plasma is involved in maintaining the stability of sperm chromatin^56^. Studies have shown that chromatin stability is high in normal men with high zinc content in their seminal plasma, but it is low in infertile men with less stable sperm chromatin^57^,^58^,^59^. Third, zinc exerts an *in vitro* effect on oxidative changes in human semen and is considered a scavenger of excessive O_2_ production by defective spermatozoa and/or leukocytes after ejaculation^60^. Fourth, Zn plays an important role in the development of testes and secondary sexual characteristics and in a few sperm physiologic functions. Zinc acts as a growth factor, an immune-regulator, and a cryoprotectant with anti-inflammatory effects, and decreased zinc levels cause hypogonadism, decreases in the testis volume, inadequate development of secondary sexual characteristics, and atrophy of seminiferous tubules, with negative effects on sperm development^61^,^62^,^63^.

In addition, some studies have suggested that zinc plays an important role in prostate health; several studies in the last decade have attempted to correlate zinc levels with semen quality parameters. Although some studies have reported that pathologic conditions of the prostate gland do not necessarily implicate interference with sperm function, other studies have also reported that, in prostatitis patients, zinc concentrations in the seminal plasma and prostatic fluid were also decreased^64^,^65^,^66^,^67^,^68^. It is known that prostatic fluid contributes greatly to the zinc content of the ejaculate. A sharp decrease in zinc in the prostatic fluid must result in a decreased zinc concentration in seminal plasma, and some studies have also reported that chronic prostatitis has negative effects on sperm parameters^69^,^70^,^71^,^72^,^73^,^74^,^75^. Thus, the zinc concentrations in prostate tissue also play an important role in sperm quality. In this meta-analysis, only Rui W *et al*. and Fuse H *et al*. discussed this factor. Some studies have suggested a relationship between zinc levels and standard variables, such as sperm motility^76^,^77^ and/or sperm count^78^, but they have shown contradictory results. Our study revealed that zinc supplementation could significantly increase the sperm volume, sperm motility and percentage of normal sperm morphology. Some studies have reported that zinc is considered one of the major factors that affect spermatozoa motility; it controls its effects by modulating the activity of the Ca^2+^ ATPase enzyme and reducing antisperm antibodies, particularly IgG. Regarding the mechanism of the influence of Zn on the semen volume, as discussed above, prostatic fluid contributes greatly to ejaculate the zinc content. In the prostate, zinc is involved in regulating the growth and apoptosis of prostate epithelial cells. The increase in prostatic fluid may contribute to the increase in semen volume. Zn was also shown to be necessary for maintaining the stability of sperm chromatin and membrane stabilization and inhibiting apoptosis for normal sperm morphology. However, the mechanism by which zinc supplementation increases the sperm quality needs further study. This meta-analysis suggested that zinc supplementation might increase male reproduction function, and these findings could open new avenues of future fertility research and treatment and could affect public health. However, this field requires further study.

There were some limitations in our study that should be considered when interpreting the results of this meta-analysis. First, the sample size of each study was relatively small, and 2,600 infertile men and 867 normal controls were investigated in all twelve studies; thus, the control group size was particularly small. Second, several studies related to the subject were excluded due to a lack of control data, means or standard deviations or the inability to obtain the full text. Third, although this meta-analysis showed that the seminal plasma zinc level decreases in infertile patients, it is not clear whether the change in the seminal plasma zinc concentration is the result of male infertility or whether the change in zinc concentration led to male infertility. Seminal plasma zinc levels also have limited predictive value because zinc is a primarily intracellular ion whose levels fluctuate according to circadian rhythm. As such, it is difficult to draw definitive conclusions concerning the clinical value of seminal zinc concentrations in male infertility.

In summary, the present study illustrated that zinc in the seminal plasma of infertile males was significantly lower than that in normal males. Zinc supplementation could significantly increase the semen volume, sperm motility and percentage of normal sperm morphology of infertile males, suggesting that zinc supplementation might increase male reproductive function. These findings could open new avenues of fertility research and treatment and could affect public health. However, further studies with larger sample sizes are needed to better elucidate the correlation between seminal plasma zinc levels and male infertility.

## Methods

### Literature search

This meta-analysis was restricted to published studies that investigated the correlation between seminal plasma zinc concentrations and male infertility and the effects of zinc supplementation on sperm parameters. Two independent reviewers searched the PubMed, EMBASE, Science Direct/Elsevier, and CNKI databases, as well as the Cochrane Library, from inception to July 2015; the language or study type was not restricted. The search terms combined text words and MeSH terms. For example, the search terms for seminal plasma zinc concentration were: ‘semen zinc concentration’, ‘semen zinc content’, ‘seminal plasma zinc concentration’, ‘seminal plasma zinc content’, ‘seminal plasma zinc level’, and ‘zinc level’. The search terms for male infertility were ‘sterility’, ‘infertility’, and ‘dysgenesis’, and the search terms for zinc supplementation were ‘zinc supplementation’, ‘added zinc’, ‘zinc supplements’, ‘oral zinc sulfate’, and ‘oral zinc gluconate’. The search terms for semen parameters were ‘sperm’, ‘spermatozoa’, ‘semen analysis’, ‘seminal parameters’, ‘sperm count’, ‘spermatozoon count’, ‘sperm motility’, ‘sperm parameters’ and ‘spermatozoon density’. All of the related articles and abstracts were retrieved. In addition, references cited within relevant reviews were retrieved by hand; only full articles were searched.

### Eligibility criteria

#### Inclusion criteria

All patients presenting for infertility evaluations had a minimum of one year of unprotected intercourse. The female partners of the selected men did not present hormonal dysfunctions, tubal obstruction or reproductive system infections. The control cases were normal men and consisted of healthy men with no history of fertility problems whose partners conceived spontaneously within 1 year of regular, unprotected intercourse. Semen samples were obtained before therapeutic interventions and were analyzed according to the World Health Organization (WHO) criteria. Semen parameters included the semen volume, sperm concentration (density), sperm motility, sperm count, sperm viability, and normal and abnormal sperm morphology percentages. Available data were extracted from the articles, and the means and standard deviations of the zinc concentrations and sperm parameters were calculated in all of the groups.

#### Exclusion criteria

Studies were excluded if they were case reports or review articles. Studies involving patients with infertility accompanied by other disorders of the urogenital system and patients who were undergoing zinc supplementation therapy were also excluded.

### Study selection and validity assessment

Two independent reviewers screened the titles and abstracts of all of the citations from the literature search. All of the relevant studies that appeared to meet the eligibility criteria were retrieved. If an ambiguous decision was made based on the title and abstract, it was necessary to analyze the full text. The final decision of eligible studies was made by reviewing the articles. Disagreements were resolved by consensus or a third reviewer.

### Data extraction and statistical analysis

Data, including demographic data (authors, year of publication, country, number and mean age of the participants) and outcome data of the seminal plasma zinc concentrations and semen parameters in all of the included studies, were extracted from the studies by three reviewers. Disagreements were resolved by consensus. Quantitative meta-analysis was performed by two reviewers using Review Manager (RevMan) software (version 5.2; the Nordic Cochrane Centre, the Cochrane Collaboration, 2012, Copenhagen, Denmark) and Stata software (version 12.0; College Station, Texas, USA). Available data were analyzed in a meta-analysis.

We pooled the standard mean differences (SMDs) of the semen zinc concentrations and sperm parameters from the included studies, which were identified with 95% confidence intervals (95% CIs). Heterogeneity was assessed by the P-value and I-square statistic (I^2^) in the pooled analyses, representing the percentage of total variation across studies. If the P-value was less than 0.1, or the I^2^-value was greater than 50%, the summary estimate was analyzed in a random-effects model. Otherwise, a fixed-effects model was applied. To estimate the stability of the meta-analysis, we conducted a sensitivity analysis. Publication bias was detected using the visual symmetry of funnel plots, with asymmetry suggesting possible publication bias. Publication bias was also assessed by Begg’s test and Egger’s test in the meta-analysis. If the P-value was less than 0.05, publication bias existed.

## Additional Information

**How to cite this article**: Zhao, J. *et al*. Zinc levels in seminal plasma and their correlation with male infertility: A systematic review and meta-analysis. *Sci. Rep.* **6**, 22386; doi: 10.1038/srep22386 (2016).

## Figures and Tables

**Table 1:** Characteristics of the included studies investigating seminal plasma zinc concentrations and male infertility.

Study	Country	Mean age (cases/controls)	Cases	Zinc supplementation	Abstinence	Assay	Semen parameters
Türk S 2014	Estonia	31/31	32		NI	NI	
Fuse H 1999	Japan	NI	14		5	AAS	
Colagar AH 2009	Iran	NI	15		NI	AAS	
Camejo MI 2011	Venezuela	33.6 ± 9.6/34.3 ± 6.4	67		3–5	XFR	
Hadwan MH 2012	Iran	NI	37	zinc sulfate 220 mg	NI	AAS	SV, STC, SM, SNM
Hadwan MH 2014	Iran	NI	60	zinc sulfate 220 mg	NI	AAS	SV, STC, SM, SNM
Akinloye O 2010	Nigeria	35 ± 1.2/36.6 ± 1.0	30		NI	AAS	
Haidar M 2013	Iran	NI	18	zinc sulfate 220 mg	NI	AAS	SV, SC, SM, SNM, SPV
Chia SE 2000	Singapore	34.8 ± 5.3/34.2 ± 4.3	107		3	AAS	
Wong WY 2002	South Africa	34.1 ± 4.1/35.3 ± 4.4	107	zinc sulfate 66 mg	NI	NI	SV, SC, SM, SNM, SPA
Li Y 2013	China	NI	58	NI	3–7	CCT	SM, SPV
Li FB 2008	China	49.3 ± 2.4/32.6 ± 2.9	63		3–7	AAS	
Liao CS 2011	China	NI	28		5	CCT	
Shi KH 2014	China	29.2 ± 2.9/30.9 ± 3.1	154		5	AAS	
Wang R 2006	China	NI	119		2–4	AAS	
Xu X 1997	China	NI	17		3–5	AAS	
Zhang DT 2003	China	NI	876	zinc gluconate 10 ml	7	AAS	SV, SPV,STC,SPA
Zheng LP 2012	China	NI	265		3–7	AAS	
Li P 2012	China	NI	500		3–7	AAS	
He Y 2011	China	NI	33		5–7	AAS	

Abbreviations: SV, semen volume; SC, sperm concentration (density); SPV, sperm viability; SNM, sperm normal morphology; SPA, sperm abnormal morphology; SM, sperm motility; STC, sperm count; AAS, atomic absorption spectrophotometry; XRF: radionuclide-induced energy dispersive X-ray fluorescence test; CCT, chemical chromatometry test. NI, not indicated in the study.

**Table 2:** Zn dosages and sperm parameters of the included studies.

	Zn dose	Sperm concentration	Semen volume	Sperm viability	Sperm normal morphology	Sperm abnormal morphology	Sperm count	Sperm motility
Hadwan MH 2012	zinc sulfate 220 mg		+		+		−	+
Hadwan MH 2014	zinc sulfate 220 mg		+		+		+	+
Haidar M 2013	zinc sulfate 220 mg	−	−	+	+			+
Wong WY 2002	zinc sulfate 66 mg	+	−		−	−		+
Li Y 2013	NI			+				+
Zhang DT 2003	zinc gluconate 10 ml	+	+		+	+		

NI, not indicated in the study; +, a significant difference between before and after zinc supplementation; −, no significant difference between before and after zinc supplementation.

**Table 3:** Egger’s test of publication bias.

Std_Eff	Coef.	Std. Err.	t	P > |t|	(95% Conf. Interval)
slope	−0.53	0.52	−1.04	0.32	−1.63 0.57
bias	−0.51	2.65	−0.19	0.85	−6.16 5.14
